# Scedosporium Apiospermum: A Rare Cause of Necrotizing Otitis Externa. Our Experience and A Review of the Current Literature

**DOI:** 10.1007/s12070-025-05395-7

**Published:** 2025-03-12

**Authors:** Eleonora Chiavarini, Luca Cerritelli, Alberto Caranti, Andrea Ciorba, Nicola Malagutti, Michela Borin, Stefano Pelucchi

**Affiliations:** 1 ENT & Audiology Department, University Hospital of Ferrara, via Aldo Moro 8, 44124 Cona, FE Italy; 2 Gruppo Otorinolaringoiatrico Della Romagna, Primus Medical Center, via Punta di Ferro, 2/c, 47122 Forlì, FC Italy

**Keywords:** Necrotizing otitis externa, External otitis, External ear pathology, Scedosporium apiospermum, Malignant external otitis

## Abstract

Necrotizing otitis externa (NOE) is a severe and progressive infection of the external auditory canal that poses significant diagnostic and therapeutic challenges, particularly when caused by rare pathogens such as *Scedosporium apiospermum*. This case report details an 84-year-old diabetic male with NOE treated with a combination of oral and topical Voriconazole after multiple ineffective antibiotic therapies. The consequent literature review identifies and analyzes 8 cases of NOE caused by *S. apiospermum*, revealing significant challenges in diagnosis and management. Diagnostic delays in NOE caused by *Scedosporium apiospermum* were common, leading to prolonged empirical antibiotic therapy in most cases. The follow-up duration was very long, with high rates of morbidity and mortality, including serious complications. Voriconazole emerged as the most effective antifungal treatment, though its use is associated with substantial risks, necessitating careful monitoring. Our findings underscore the importance of considering fungal etiologies in persistent NOE and emphasize the need for repeated ear swabs and targeted therapy to improve patient outcomes. The results highlight a critical gap in standardized diagnostic and therapeutic protocols, advocating for more rigorous evaluation and management strategies in cases of suspected *Scedosporium* infections.

## Introduction

Necrotizing otitis externa (NOE) is a severe, progressive, and potentially life-threatening infection of the external auditory canal (EAC).

The symptoms of NOE may resemble those of simple otitis externa, such as itching, aural fullness, otalgia, and otorrhea, but it typically does not respond to conventional antibiotic therapies and exhibits an erosive nature. NOE can erode the bony walls of the EAC and extend to adjacent structures such as the skull base. This aggressiveness is usually secondary to comorbidities such as diabetes mellitus or immunodeficiency [1].

NOE is a complex infection that poses challenges in diagnosis and management. Radiological studies, including computed tomography (CT), magnetic resonance imaging (MRI), and nuclear imaging, are valuable tools for diagnosis and follow-up. The most commonly identified pathogen in this infection is *Pseudomonas aeruginosa*, while fungal etiologies are rare [2]. Among fungal pathogens, *Aspergillus* and *Candida* species are frequently encountered. Although less common, *Scedosporium apiospermum* (*S. apiospermum*) has also been recognized as a causative agent of NOE, with a very limited number of cases reported in the literature.

*S. apiospermum* is a filamentous fungus found in soil, sewage, and polluted water and can act as a saprophyte in the external auditory canal.

The treatment of *Scedosporium* infections is challenging due to resistance to many antifungal agents [3]. The most effective antifungal for *Scedosporium* infections appears to be Voriconazole, a second-generation triazole available in both oral and intravenous formulations. However, the widespread use of this antifungal has been limited due to the high incidence of adverse effects involving the liver, central nervous system, and kidneys [4, 5].

In this article, we reported a case of NOE caused by *S. apiospermum* in a diabetic patient treated with topical and oral Voriconazole. Subsequently, we compared this case with the few instances documented in the literature, examining factors such as delays in diagnosis, types of medical and surgical treatments, and associated complications.

## Case Report

We report the case of an 84-year-old Caucasian man with poorly controlled type II diabetes mellitus and bilateral hearing aids for sensorineural hearing loss. He was admitted to our clinic with persistent left-sided otalgia and otorrhea lasting approximately one month.

The initial examination revealed left purulent otorrhea, for which an ear swab was collected for culture, and inflammatory stenosis of the external auditory canal (EAC), for which a medicated endoaural gauze soaked with topical ciprofloxacin was placed. The patient was treated with oral therapy consisting of Amoxicillin-clavulanate (875 mg + 125 mg, three times daily for 10 days).

Although the swab result was negative, a reassessment after 24 days showed no improvement. Consequently, second-line empirical therapy with Ciprofloxacin 500 mg (two tablets daily for 15 days) was initiated, the oral hypoglycemic therapy was optimized (as recommended by a specialist), a second culture swab (also negative) was performed, and a CT scan was ordered. The CT revealed tympanic effusion, mastoid involvement, and bony erosion of the supero-anterior wall of the EAC, leading to a diagnosis of necrotizing otitis externa (NOE).

The dose of Ciprofloxacin was subsequently increased to 750 mg twice daily for 30 days, as recommended by an infectious disease specialist. However, the infection continued to progress, with the development of an EAC polyp and swelling of the left parotid gland, prompting an MRI. The MRI confirmed inflammation of the parotid, masticatory, and temporal muscles, without evidence of abscesses.

The lack of response to therapy led to the patient’s hospitalization and initiation of third-line therapy with Piperacillin/Tazobactam and Linezolid.

Only 70 days after the patient’s initial evaluation did a third ear swab return positive, identifying *Scedosporium apiospermum* as the causative pathogen. A leukocyte-labeled scintigraphy was performed, which was negative for temporal and skull base osteomyelitis. Medical therapy was modified to oral Voriconazole 200 mg twice daily, and local therapy was introduced, consisting of Triamcinolone acetonide injections (40 mg/mL) and topical applications of Voriconazole (aqueous solution 1%, 5 drops four times daily). Glycemic control was improved through the initiation of insulin therapy. Following these adjustments, the EAC edema began to decrease along with the otorrhea.

The patient was discharged after achieving a negative ear swab, continuing oral and topical Voriconazole after EAC disinfection with Betadine solution.

During subsequent outpatient evaluations, EAC edema was still present, along with indirect signs of retrotympanic effusion observed through tympanometry and microscopy. Consequently, a surgical solution was chosen to improve ear ventilation and enhance the effectiveness of medical therapy. A meatoplasty and transtympanic drainage were performed. Follow-up evaluations showed progressive improvement in otorrhea, leading to complete resolution 205 days after the patient’s initial presentation.

Ultimately, after multiple courses of antibiotic therapy, four months of oral Voriconazole, nine weeks of topical Voriconazole, and seven Triamcinolone acetonide injections, the patient was deemed cured.

## Discussion

Necrotizing otitis externa (NOE) is a severe and potentially life-threatening infection of the temporal bone that requires adequate and prolonged medical therapy. If untreated, the infection can extend beyond the temporal bone, eroding it and potentially reaching the meningeal plane, causing severe cerebral complications such as meningitis, meningoencephalitis, and brain abscesses [1].

NOE is generally a bacterial infection, with *Pseudomonas aeruginosa* being the most common pathogen. However, fungi such as *Aspergillus* and *Candida* species can also cause this condition [2]. The occurrence of NOE caused by *Scedosporium apiospermum* is exceptionally rare, with very few cases reported in the literature, making diagnosis and treatment challenging.

We therefore conducted a review of the current literature using the following keywords on PubMed: “Scedosporium apiospermum” AND “necrotizing otitis externa”; “Scedosporium apiospermum” AND “skull base osteomyelitis”; and “Scedosporium apiospermum” AND “otitis.” This search yielded 28 articles. We excluded duplicates and all articles that did not pertain to cases of NOE, focusing instead on those addressing simple external or middle ear infections, as well as all cases of skull base osteomyelitis not of otogenic origin, as depicted in the PRISMA flow chart [6]. Ultimately, we included 8 relevant studies, whose characteristics are listed in Table 1.

**Table 1:** The table summarizes the 8 studies included in our review, along with the main anamnesis data of the patients

Authors	Otomicroscopy EAC	Presence of Mastoid Effusion (Imaging)	Complications	Surgery
Yao M et al. [7]	Inflammatory polyp	Yes	VII nerve paralysis Confusional state	Mastoidectomy
Vasoo S et al. [8]	Inflammatory Polyp Granulation tissue	No	VII nerve paralysis IX nerve paralysis XII nerve paralysis	No
Huguenin A et al. [9]	Otorrhea EAC stenosis	Yes	None	No
McLaren O et al. [10]	Otorrhea Hyperemia of the EAC walls	Yes	VII nerve paralysis Skull base osteomyelitis	No
Jalava-Karvinen P et al. [11]	Inflammatory polyp Granulation tissue Ulcer of the duct walls Otorrhea with fungal hyphae	No	VI nerve paralysis Cerebral abscess Meningitis Internal carotid aneurysm	FESS
Doss M et al. [12]	Otorrhea EAC stenosis	Yes	Cerebral stroke	No
Fuster-Escrivá B et al. [13]	Otorrhea Granulation tissue	No	VII nerve paralysis Cardiovascular arrest	No
Hussain SZM. Haq II et al. [14]	Otorrhea Granulation tissue Inflammatory polyp	Yes	VII nerve paralysis	Polypectomy + Canaloplasty
Our case	Otorrhea Inflammatory polyp EAC stenosis	Yes	Extension of the infection to the parotid and masticator muscles (not abscess)	Meatoplasty + Ventilation tube

All documented cases in the literature involve male patients, 62.5% of whom are older than 75 years.

We know that *S. apiospermum* has a uniform worldwide geographic distribution and is usually responsible for cutaneous infections that, in immunocompromised individuals, may become systemic. Not surprisingly, as documented in Table 2, all cases of NOE reported in the literature occurred in patients with predisposing conditions such as diabetes mellitus or AIDS, including our patient.

**Table 2:** The table outlines the clinical data related to the 8 case studies included in our review, as well as the case reported by us in this article

Authors	Risk factors	Duration of pre-visit symptoms	Otomicroscopy EAC	Radiologic studies			Duration complete follow-up	Outcome
				CT scan	MRI	Nuclear Medicine Imaging		
Yao M et al. [7]	AIDS	6 Months	Inflammatory polyp	No	Yes (1 exam)	No	Not specified	Death
Vasoo S et al. [8]	Diabetes	1 Month	Inflammatory Polyp Granulation tissue	Yes (1 exam)	Yes (3 exams)	No	11 Months	VII nerve paralysis
Huguenin A et al. [9]	Hearing aid	6 Months	Otorrhea EAC stenosis	Yes (2 exams)	Yes (1 exam)	No	11 Months	Complete recovery
McLaren O et al. [10]	Diabetes	3 Weeks	Otorrhea Hyperemia of the EAC walls	Yes (2 exams)	No	Yes (1 SPECT)	9 Months	Conductive hearing loss
Jalava-Karvinen P et al. [11]	Diabetes renal failure Chronic Steroid	Not Specified	Inflammatory polyp Granulation tissue Ulcer of the duct walls Otorrhea with fungal hyphae	Yes (1 exam)	Yes (4 exams)	No	7 Months	Death
Doss M et al. [12]	None	Not Specified	Otorrhea EAC stenosis	Yes (3 CT + 2 angio-CT)	Yes (4 exams)	No	4 Months	Stroke outcomes
Fuster-Escrivá B et al. [13]	Diabetes Vasculopaty	Not Specified	Otorrhea Granulation tissue	Yes (2 exams)	No	No	3 Months	Death
Hussain SZM. Haq II et al. [14]	Diabetes	2 Months	Otorrhea Granulation tissue Inflammatory polyp	Yes (1 exam)	Yes (2 exams)	No	Not specified	Complete recovery
Our case	Diabetes Hearing aid	1 Month	Otorrhea Inflammatory polyp EAC stenosis	Yes (2 exams)	Yees (3 exams)	Yes (1 Scintigraphy)		

Furthermore, it is important to note that the development of external otitis caused by *S. apiospermum* typically occurs when constant drainage maintains moisture in the canal, as in chronic otitis; when the canal is obstructed by desquamating debris, as in dermatitis; or by external devices, such as hearing aids (as in our patient’s case) [3].

The difficulty in correctly identifying the causative agent is highlighted by the fact that most patients reported typical symptoms of external otitis for at least one month before evaluation. In the American Academy guidelines, initial topical treatment, including antibiotics, is recommended [15]. However, the various topical antibiotic therapies administered at symptom onset, the abundant presence of natural saprophytes on the skin, and the slow in vitro growth rate of *S. apiospermum* [9] often result in a series of negative or inconclusive ear swabs. In our dataset, 2 patients (22%) had a positive first swab [7, 9]. More frequently, the second swab was positive (5/9 patients); in our case and in that of Jalava-Karvinen P et al., the etiological diagnosis was made only with the fourth ear swab [11]. The long time required for diagnosis results in significant delays in initiating targeted antifungal therapy, compounded by the fact that most NOE cases are due to *P. aeruginosa* infections, leading to empirical antibiotic therapy. Table 3 clearly shows how, in all cases, including ours, patients were initially treated with empirical antibiotic therapy.

**Table 3:** The table outlines the data related to the medical treatments applied in the 8 case studies from our review and in the case reported by us in this article

Authors	Number antibiotics (Empiric)	Number first scedosporium positive swab	Delay in starting voriconazole therapy	Duration voriconazole therapy
Yao M et al. [7]	2	1st	Not specified	Not specified
Vasoo S et al. [8]	5	2nd	5 Months	6 Months
Huguenin A et al. [9]	11	1st	9 Months	2 Months
McLaren O et al. [10]	4	2nd	3 Months	6 Weeks
Jalava-Karvinen P et al. [11]	4	4th	3 Months	4 Months
Doss M et al. [12]	2	2nd	1 Week	3 Months
Fuster-Escrivá B et al. [13]	1	2nd	2 Months	1 Month
Hussain SZM. Haq II et al. [14]	8	2nd	Not specified	Not specified
Our case	5	4th	10 Weeks	4 Months

Following microbiological diagnosis, the challenge lies in determining the most appropriate therapy. Literature indicates that in vitro susceptibility testing of *Scedosporium apiospermum* isolates to various antifungal classes (including azoles, polyenes, allylamines, pyrimidines, and echinocandins) reveals that Voriconazole exhibits the highest antifungal efficacy [3]. In contrast, Amphotericin B, other polyenes, and 5-flucytosine demonstrate lower efficacy. It is noteworthy that Voriconazole has been shown to cross the blood-brain barrier and does not rely on meningeal inflammation for its efficacy, making it suitable for treating NOE and its complications, such as brain abscesses. However, the use of Voriconazole is not without risks, as it is associated with a higher frequency of adverse events, including neurotoxicity, visual disturbances, hepatotoxicity, and potential nephrotoxicity [4, 5]. Such side effects may necessitate discontinuation of Voriconazole and switching to alternative antifungal agents, which may have reduced efficacy against *S. apiospermum*.

Our review of the literature showed (Table 3) that in 5 out of the 8 cases examined, there was a delay of at least two months before initiating Voriconazole therapy following the first specialist evaluation.

Another controversial aspect in the literature concerns the duration of antifungal therapy with Voriconazole. Prolonged treatment is often necessary; in fact, half of the cases examined required at least four months of therapy, exposing patients to a high risk of developing hepatic, cerebral, and renal side effects.

**Fig. 1:**
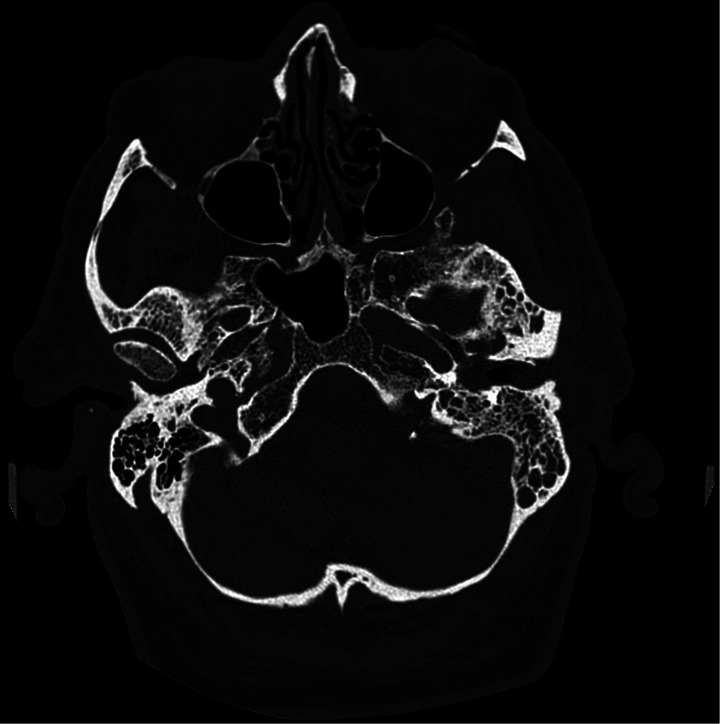
CT scan showing mastoiditis afflicting left ear

In a few cases, surgical management was associated with medical therapy, implemented either for resolving the infection in the external auditory canal (EAC) or for draining the mastoid. Huguenin A. and colleagues reported in a mini-review of 15 cases that 60% (9/15 cases) exhibited extension to adjacent bones or soft tissues, with mastoiditis present in 53.3% of cases (8/15 cases) [9]. In our collected cases, 5 cases of external otitis were accompanied by tympanic effusion (Table 4). Our patient also presented with otitis media and mastoiditis (the CT scan showing mastoiditis is illustrated in Fig. 1). However, we did not perform surgical drainage with mastoidectomy, as in the case published by Yao M. et al., but only a transtympanic ventilation tube was placed [7]. Additionally, in our patient and in the case presented by Hussain S.Z.M. et al., surgical debridement of the EAC was performed [14].

**Table 4:** The table outlines the data related to the surgical treatments applied and the complications observed in the 8 case studies from our review and in the case reported by us in this article

Authors	Year	Number of patient	Patient age	Sex
Yao M et al. [7]	2001	1	21	Male
Vasoo S et al. [8]	2007	1	51	Male
Huguenin A et al. [9]	2015	1	84	Male
McLaren O et al. [10]	2016	1	79	Male
Jalava-Karvinen P et al. [11]	2016	1	48	Male
Doss M et al. [12]	2018	1	88	Male
Fuster-Escrivá B et al. [13]	2019	1	77	Male
Hussain SZM. Haq II et al. [14]	2023	1	81	Male

Regarding radiological imaging, CT scan is the technique of choice for evaluating EAC bone erosion, while MRI or angio-MRI are valuable for assessing the extent of infection to adjacent soft tissues. Moreover, in cases with neurological symptoms, it is critical to exclude the possibility of brain abscesses and intracranial vessel thrombosis. It is also worth noting that, in all evaluated clinical cases, these radiological examinations were used to assess therapeutic responses.

Nuclear medicine techniques are less frequently used but can be employed to confirm or rule out skull base osteomyelitis, as demonstrated in the case reported by McLaren and Potter, as well as in the patient described in this report, where similar diagnostic investigations were conducted [10].

Despite the undeniable utility of radiological studies, there is currently no standardized protocol for imaging modalities or the timing of radiological follow-up in NOE cases. In the cases collected in this review, as in our patient, the overall follow-up duration for patients is significantly prolonged, averaging 8 months. This condition carries a considerable risk of mortality or sequelae, affecting 6 out of 9 patients, with 50% having a poor prognosis, and only 3 patients achieving complete recovery.

The patient’s pre-existing fragility, delays in diagnostic procedures, and subsequent challenges in managing *S. apiospermum* NOE contribute to an increased risk of complications, which can even lead to fatal outcomes. As indicated in Table 4, most patients presented with paresis of one or more cranial nerves; in fact, the most frequently observed complication is VII cranial nerve palsy (66%). In terms of more severe complications, the patient described by Doss M. et al. developed internal carotid thrombosis, leading to cerebral stroke [12]. Furthermore, three of the eight patients in our review had a poor prognosis: two patients died due to cerebral complications (one from a brain abscess and the other from meningitis), while one patient experienced cardiac arrest. Conversely, our clinical case and that reported by Huguenin A. and Noel V. et al. were the only cases that did not report complications [9].

We hypothesize that the absence of complications in our case is primarily attributable to the use of topical Voriconazole therapy, a therapeutic approach not employed in any other clinical case reported in the literature.

## Conclusion

*Scedosporium apiospermum* is a rare cause of necrotizing otitis externa, characterized by a challenging etiological diagnosis and risky therapeutic management, full of potential toxicity. Its rarity, combined with the difficulty of rapid recognition and effective management, poses a serious risk of severe complications. Therefore, it is crucial to consider fungal etiology in cases where multiple antibiotic therapies have proven ineffective.

Furthermore, is essential to perform ear swabs and eventually to repeat them if they are negative and/or if there is no clinical improvement. The isolation, the proper identification, and susceptibility testing of the fungal agent are fundamental steps in optimizing the treatment of *Scedosporium* infections.

## Data Availability

All data generated or analysed during this study are included in this article. Further enquiries can be directed to the corresponding author.
